# Development and Validation of a Controlled Vocabulary: An OWL Representation of Organizational Structures of Trauma Centers and Trauma Systems

**DOI:** 10.3233/SHTI190252

**Published:** 2019-08-21

**Authors:** Joseph Utecht, Jane Ball, Stephen M. Bowman, Jimm Dodd, John Judkins, Robert T. Maxson, Rosemary Nabaweesi, Rohit Pradhan, Nels D. Sanddal, Robert J. Winchell, Mathias Brochhausen

**Affiliations:** a Department of Biomedical Informatics, University of Arkansas for Medical Sciences, Little Rock, Arkansas, USA,; b American College of Surgeons, Chicago, Illinois, USA; c Department of Health Policy and Management, University of Arkansas for Medical Sciences, Little Rock, Arkansas, USA; d Department of Biology, University of Pennsylvania, Philadelphia, Pennsylvania, USA; e Department of Surgery, University of Arkansas for Medical Sciences, Little Rock, Arkansas, USA; f Department of Pediatrics, University of Arkansas for Medical Sciences, Little Rock, Arkansas, USA; g Department of Surgery, Weill Cornell Medical College, New York, New York, USA

**Keywords:** Trauma Centers, Vocabulary, Controlled, Biomedical Ontologies

## Abstract

In trauma care and trauma care research there exists an implementation gap regarding a consistent controlled vocabulary to describe organizational aspects of trauma centers and trauma systems. This paper describes the development and evaluation of a controlled vocabulary for trauma care organizations. We give a detailed description of the involvement of domain experts in the domain analysis workflow and the authoring of definitions and additional term descriptions. Finally, the paper details the evaluation methodology to assess the initial version of the controlled vocabulary. The results of the evaluation show that our development process yields terms most of which find approval from domain experts not involved in the development. In addition, our evaluation tools resulted in valuable domain expert input to optimize the controlled vocabulary.

## Introduction

Since the 1970s, the American College of Surgeons (ACS) has worked to establish and refine criteria that set the standards for trauma centers. Due to the inherent difficulty in obtaining reliable outcome data, these standards have focused largely on measures of structure and process. The most recent standards were published in *Resources to Optimal Care of the Injured Patient (2014)* [1]. In 2008, the ACS established the Trauma Quality Improvement Program (TQIP) to enable trauma centers to measure and compare their risk-adjusted patient outcomes to similar organizations [2,3]. Though the TQIP program continues to grow, at this time the relationship between structure and process measures and clinical outcomes remains elusive. Further, beyond the standards themselves, the ways in which the particular attributes of trauma centers, and at a larger scale, of regional trauma systems, contribute to optimal patient outcomes have not yet been identified or measured.

While the trauma center standards have paved the way for a consistent and reusable terminology, there still exists an implementation gap in providing and using a well-structured controlled vocabulary to describe the structure of trauma centers and trauma systems. Having such a resource will facilitate transferring knowledge and experience from one trauma center or trauma system to another. In addition, controlled vocabulary also will facilitate comparison of organizational structures and procedures both nationally and internationally.

CAFÉ (Comparative Assessment Framework for Environments of Trauma Care) is an NIH-funded project that aims to collect detailed information on the particular organizational attributes of trauma systems and trauma centers. Ultimately, the goal is to link that data to clinical outcome data to identify those organizational attributes of trauma centers and trauma systems that are of high impact on patient outcomes. The project creates a web-based service that allows an interested individual to enter data about their trauma center or trauma system and to conduct an anonymous self-assessment of the organizational structures of their trauma center or trauma system. The first step to enable such a comparison is to provide a common controlled vocabulary covering all relevant aspects of trauma center and trauma system management. Preliminary CAFÉ project work that leveraged natural language processing to create lists of relevant terms from published abstracts pertinent to the domain was described in a previous publication [4]. In this paper we report the workflow of creating the initial version of the vocabulary in close collaboration. In addition, we describe our evaluation methodology, describe the results of the evaluation, and draw conclusions on whether the development methodology of the controlled vocabulary has been successful. For the purpose of the CAFÉ project, the controlled vocabulary needs to exist as a Web Ontology Language (OWL2) to be used with other semantic web technologies. The general outline of the project plan was published elsewhere [4].

## Methods

The first step necessary for developing the CAFÉ system was the domain analysis of organizational structures of trauma centers and trauma systems. A domain analysis for the purpose of developing a controlled vocabulary consists of identifying the relevant terms to represent the domain of interest and providing definitions for those terms. For the CAFÉ project, that meant collecting relevant terms to represent and analyze the organizational structure of trauma centers and trauma systems. Since our aim is to create an ontology that follows the OBO Foundry principles, it was prudent to use the OBO Foundry suggested form of definition, the genus-differentia (http://www.obofoundry.org/principles/fp-006-textual-definitions.html) form. In the OBO Foundry community, definitions of that form are frequently provided using the IAO:definition annotation property (http://purl.obolibrary.org/obo/IAO_0000115). In addition to the *genus-differentia definition* being an OBO Foundry requirement, these definitions are also particularly useful when creating a taxonomy for the controlled vocabulary since these definitions always refer to the parent term of the term being defined.

Earlier work on OBO Foundry ontologies has informed the project leads for ontology development that domain experts and potential users often find the *genus-differentia definition* a poor representation of how they view the domain and communicate about its phenomena. Hence, we decided to follow the advice of Hogan et al. [5] to provide definitions that are easily accessible for domain experts using an annotation property different from the IAO:definition. To allow that, we created the OOSTT user-centered description annotation property (http://purl.obolibrary.org/obo/OOSTT_00000030).

In addition, we also created the annotation property “CAFE application label” for cases where the label that was created to follow naming conventions in ontology development would be a suboptimal label to display to the user of the system (http://purl.obolibrary.org/obo/OOSTT_00000043).

The effect of this consideration on our domain analysis is that we are collecting one RDFS:label, one IAO:definition and, in cases where the *genus-differentia definition* is deemed hard to parse for domain experts, one OOSTT user-centered description.

After the CAFÉ project was funded by the National Institute of General Medical Science (NIGMS) in spring 2015 (R01GM111324), a weekly (later bi-weekly) expert call was started to identify relevant terms to assess the organizational structure of trauma centers and trauma systems and, in future work, its relationship to patient safety and outcomes. The committee consisted of two trauma surgeons, both bearing roles in the American College of Surgeons (ACS) Committee on Trauma (COT); two representatives of the COT with experience in trauma center verification and trauma system consultation; two researchers with experience in analyzing data on trauma care, including data from trauma centers and trauma systems; and one expert in organization analysis. To accomplish the domain analysis part of the project, a multi-step process was created during which the members of our expert group reviewed material from the ACS COT and added material based on their experience to identify the relevant terms and provide user-centered descriptions for those. The goal of this process was the implementation of our controlled vocabulary in the OWL language.

Step 1: *Domain set of terms*. In the first step, the expert group agreed on a set of terms necessary to cover the domain of organizational structures of trauma centers and trauma systems. This step used the American College of Surgeons’ *Resources for Optimal Care of the Injured Patient, Sixth Edition* from 2014 as a starting point [1]. The experience of the domain experts regarding the practical implementation of trauma center verification, trauma system consultation, and quality improvement on trauma, in general, informed the final decision on which terms to include.
Step 2: *Preliminary descriptions provided*. Once the initial set of terms was agreed upon by the domain expert group, preliminary descriptions of each term were provided by the domain experts. The goal was to capture a description that the domain experts were able to agree upon regardless of whether the description met the requirements of an OBO Foundry definition.
Step 3: *Preliminary descriptions turned into genus-differentia definitions.* Based on the descriptions, the CAFÉ OWL implementation team suggested *genus-differentia definitions*. In doing so, the OWL implementation teams thought about definitions and descriptions in a set-theoretical way. We assumed that for each definition/description there is a set of entities for which that definition/description is true. In creating a *genus-differentia definition* for a description it is crucial that the former is true for the same set of entities as the latter. In other words, that the p *genus-differentia definition* has the same extension as the initial description authored by the domain experts. In many cases, this process involved iterative communication between the domain experts and the ontology developers to a) achieve a clear understanding of the intended meaning and b) ensure congruency between the preliminary description and the *genus-differentia definition*.
Step 4: *OOSTT user-centered description created*. Based on whether the domain experts felt that the *genus-differentia definition* was useful for the target audience or not, OOSTT user-centered definitions were created where usefulness for the target audience was in doubt. To the extent the delimitation of the CAFÉ domain expert group agreed that the *genus-differentia definitions* are useful to the target audience was one of the parameters that we plan to assess with our evaluation approach. For the terms that have both a OOSTT user-centered description and a *genus-differentia definition* the same kind of congruency as described in Step 3 was required.
Step 5: *Approval of terms, definitions and OOSTT user-centered descriptions by domain experts*. Our aim was to submit all final terms, their definitions, and their OOSTT user-centered descriptions to another round of expert approval. While this strategy worked for many terms, restrictions created by our timeline limited another approval round for all terms.
Step 6: *OWL implementation*. Finally, all terms were included in the OWL file step-by-step during the rollout of the OOSTT ontology, which started in 2015 and was described in more detail in a previous publication [4]

**Figure 2 -:**
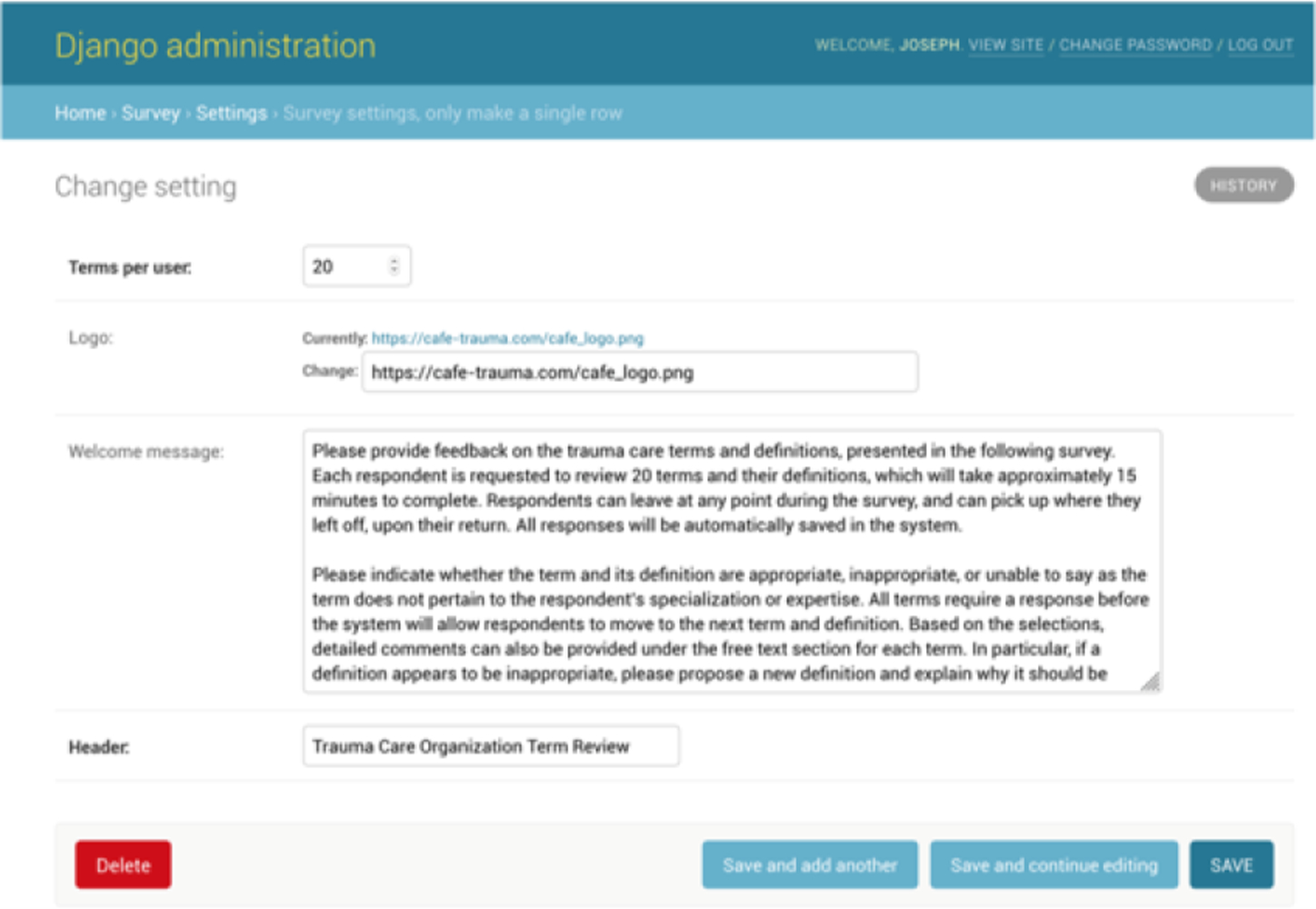
Term Tool Configuration

To help determine the quality and acceptance by our target audience of the terms and definitions created, we conducted a survey with feedback. A new surveying tool was created for the task. Our goal for this new tool was to show a small number of random terms and definitions to members of our target audience while minimizing inconvenience to them. This tool also needed to ensure a balanced number of reviews for each term and easily allow us to load the up-to-date version of the 216 terms and definitions. The term survey tool (https://github.com/cafe-trauma/term_survey) was built over two days in Python using the Django Framework. To ensure current terms and definitions could easily be loaded, the tool was built with the functionality to parse an OWL file for OWL classes from which the RDFS:label would be used for the “term” and the URI of an annotation would be passed to query for the “definition”. As mentioned above, we used a custom annotation property for “User Centered Definitions.” After the terms and definitions were loaded, an administrator set a welcome message, a logo, and a number of terms for each respondent to review (Figure 2).

**Figure 3 -:**
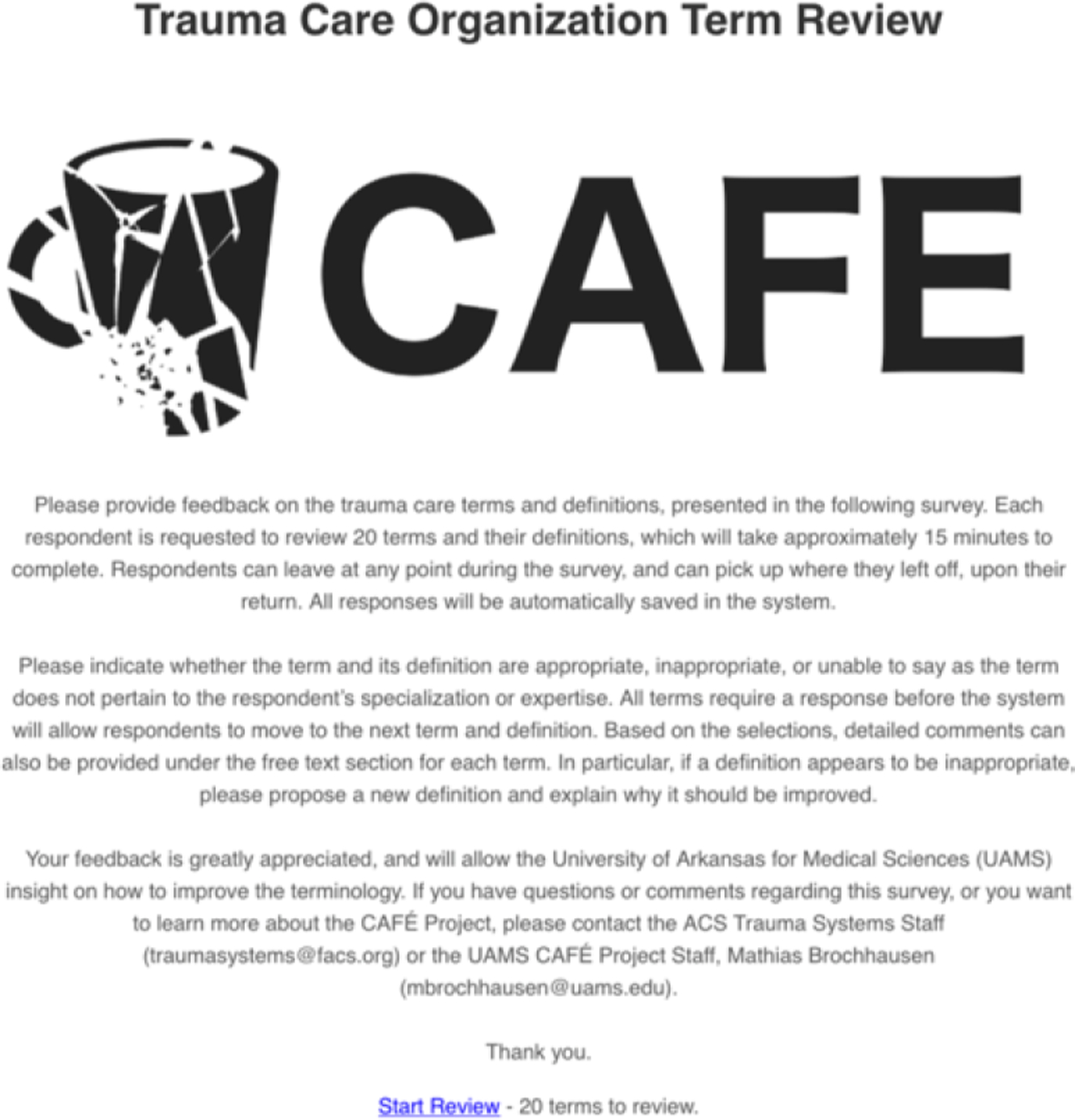
Welcome Page

**Figure 4 -:**
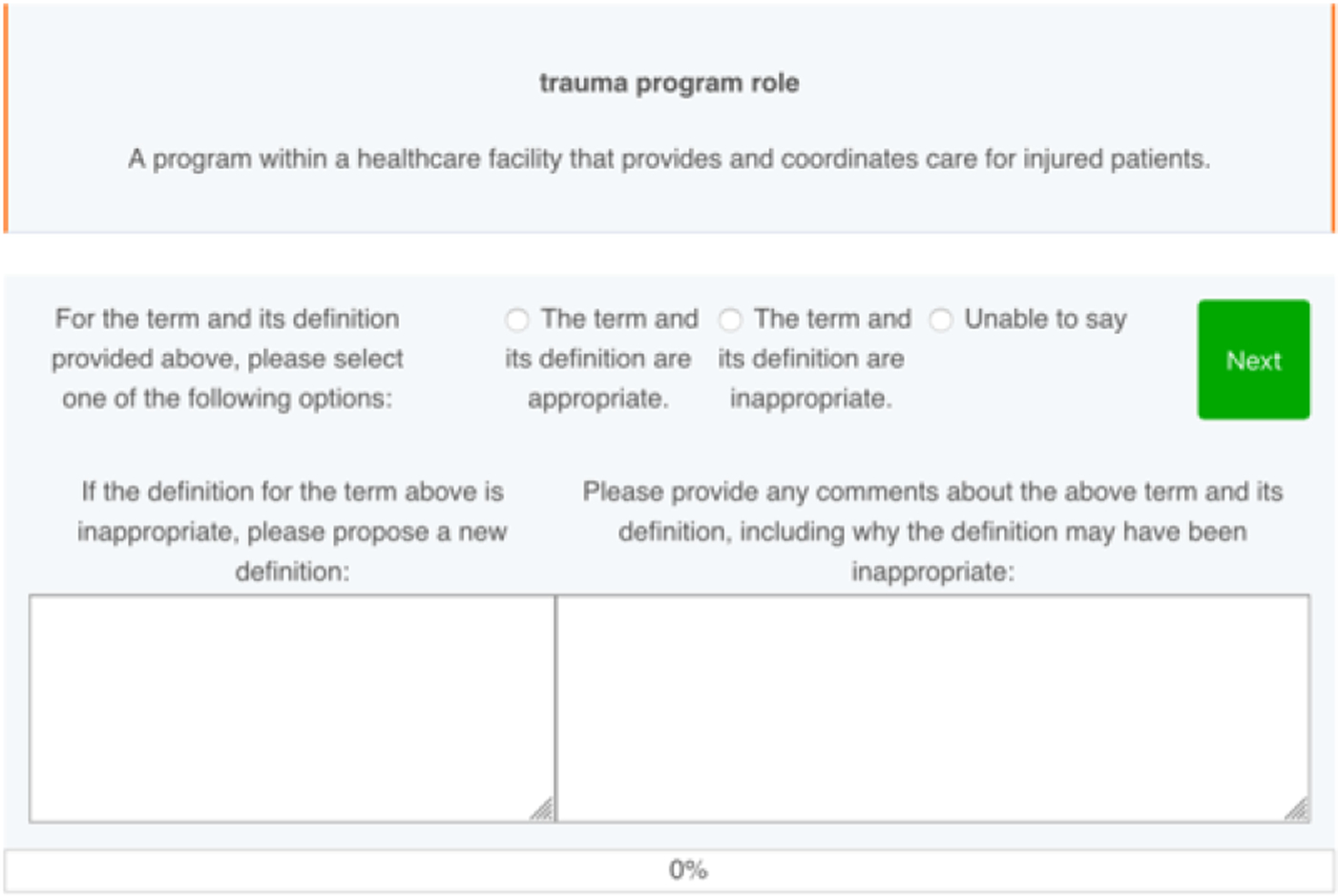
Term Review

**Figure 5 –:**
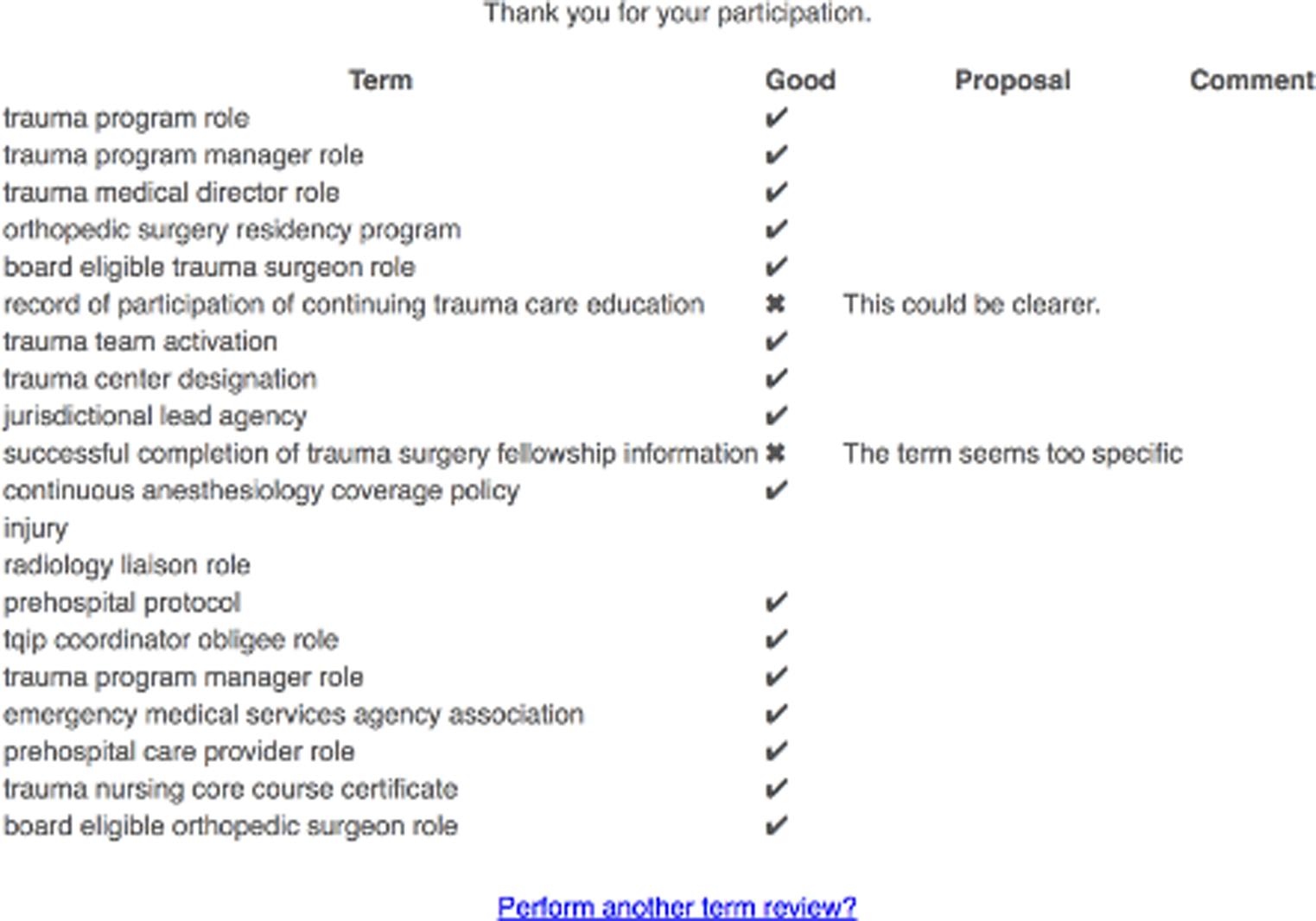
Survey Completion Summary

At this point the survey was ready. To distribute it to respondents we emailed a link to the landing page of the survey, where the respondent was greeted by the welcome message and could begin reviewing terms (Figure 3). We distributed the survey to 75 members of the ACS COT. The terms for each respondent to review were not preselected. Each time a respondent attempted to review a new term the tool would query for the current list of terms with the lowest number of reviews and return a random term from this list, ensuring an equal coverage of reviews for our terms. On the term review page, a respondent was presented with a term, its definition, radio buttons to indicate the acceptability of a definition, and space to provide feedback (Figure 4). After clicking to move to the next term, a respondent’s feedback was immediately saved to the server and a new randomly selected term was presented. The term review page also had a progress bar to indicate the number of terms remaining for review. After giving their initial feedback, a cookie was created in the respondent’s browser that tied them to their session of terms in case they closed the browser before finishing. Since we saved feedback after each term we still had feedback even if the respondent did not finish the requested number of term reviews. After reviewing the assigned 20 terms and definitions, the respondent was shown a summary screen with all of their feedback and given the choice to edit any of their feedback or to clear their session and review more terms if so inclined (Figure 5).

## Results

**Figure 6 -:**
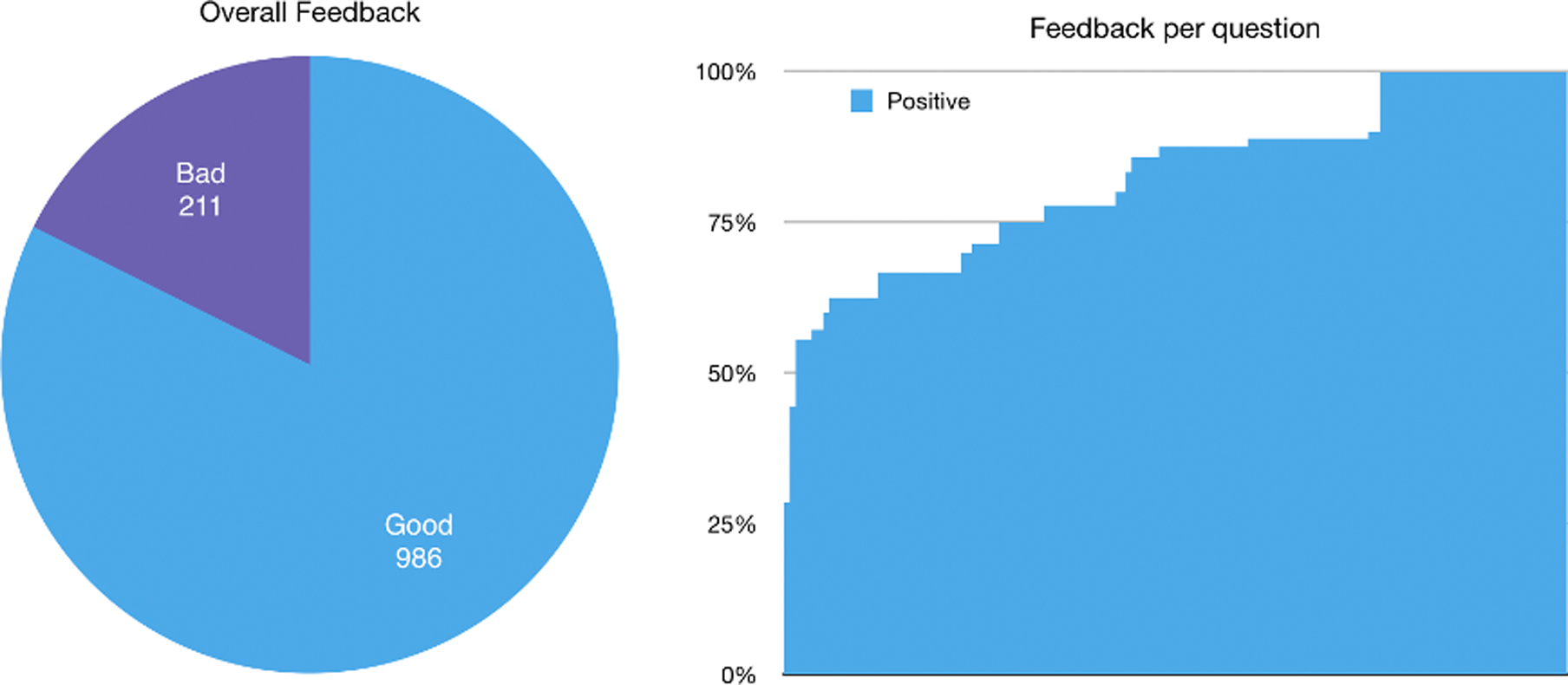
Survey Feedback

Using our newly built survey tool, we received a large amount of good feedback about our terms. The survey was distributed to 75 people in the trauma care domain. We received at least one term review from 74 people. In total, our 142 terms and definitions received 1,197 reviews. That response rate allowed us to have, on average, eight different people review each term and definition. Of the 1,197 individual reviews 986 of them considered the term and definition suitable (Figure 6). The reviewers largely agreed with each other when reviewing the same terms both positively and negatively. Our highest rated terms, such as “trauma program leadership,” “hospital governing body role,” and “trauma program” received uniformly good feedback, while the lowest rated terms, “trauma nursing evaluator obligee role,” “trauma quality improvement and patient safety program lead role,” and “emergency medical services provider association” were negatively reviewed by multiple people. Overall, 103 or 72% of our total terms received positive feedback from over 75% of reviewers (Figure 6).

The responses and comments were provided to the ontology developers who reviewed the comments and aimed to correct the issues pointed out by the domain experts. For the lowest rated terms, this included a complete revision of the definition, as in the case of “emergency medical services provider association,” which was updated based on reviewer feedback^1^. The most reported problem with “trauma quality improvement and patient safety program lead role” was that the label was singular, while the definition implied plural. The definition was changed to better reflect the label^2^. The label “trauma nursing evaluator obligee role” was critized for the use of uncommon language, namely “obligee role.” Since that class did not have a CAFÉ application label, we added the CAFÉ application label “trauma nursing evaluator”^3^.

## Discussion

The results presented in this paper provide insight into two main areas: a) domain analysis and definition authoring in a clinical domain and b) providing a novel resource for trauma care and trauma research to facilitate comparison between trauma centers and trauma systems by creating a controlled vocabulary.

The current evaluation of the initial version of the domain analysis yielded primarily positive results. Not only did participants rate most terms as “good,” they also provided insightful and highly actionable comments to make the terms and definitions more useful or correct. The curators of the controlled vocabulary have already incorporated multiple improvements based on reviewer comments and are currently working to finalize that process. At this point in time, the survey has only been distributed to trauma care experts affiliated with the American College of Surgeons. We are planning to distribute the survey to clinical staff in trauma management in order to gather their input on these terms.

Larger, international evaluations of the vocabulary are planned for 2019. Distributing the survey to experts from diverse healthcare systems will provide interesting insights into which terms are useful and which definitions are shared across healthcare systems.

While efforts to improve terminologies to advance trauma care and trauma research are not uncommon [6,7,8], literature searches conducted on PubMed reveal an astonishing lack of effort to create terminologies or controlled vocabularies relevant to trauma care in general and its organization. Oliver and Walter [9] point out the problems arising from the lack of a common terminology in trauma research related to preventable death. Searching PubMed for [“trauma system” terminology], [“trauma center” terminology], [“trauma system” vocabulary], [“trauma center” vocabulary], yields 38 distinct papers, only one of which specifically deals with the problem of providing a terminology/vocabulary for organizational structures in trauma systems and trauma centers. Notably, that paper is also the output of the CAFÉ project [4]. So, while there is an established field of work on terminologies and classifications for specific types of trauma and a corpus of research on outcome measures, there is not much effort to provide a unified language to describe the organizational structure of trauma centers and trauma systems.

## Conclusions

While some of the terms still required additional refinement, a vast majority of the terms was deemed “good” by a large group of domain experts working in collaboration with the American College of Surgeons. Based on these results we conclude that our methodologies for domain analysis and creation of definition and user-centered descriptions have successfully created an initial version of a controlled vocabulary for the ACS community and the trauma care community in the U.S.

We also believe that the number of targeted and useful comments we received from the domain experts participating in our survey highlights the fact that dividing review tasks into small portions and distributing them over a larger number of participants is a valuable approach to encourage expert feedback.

## Figures and Tables

**Figure 1 –:**
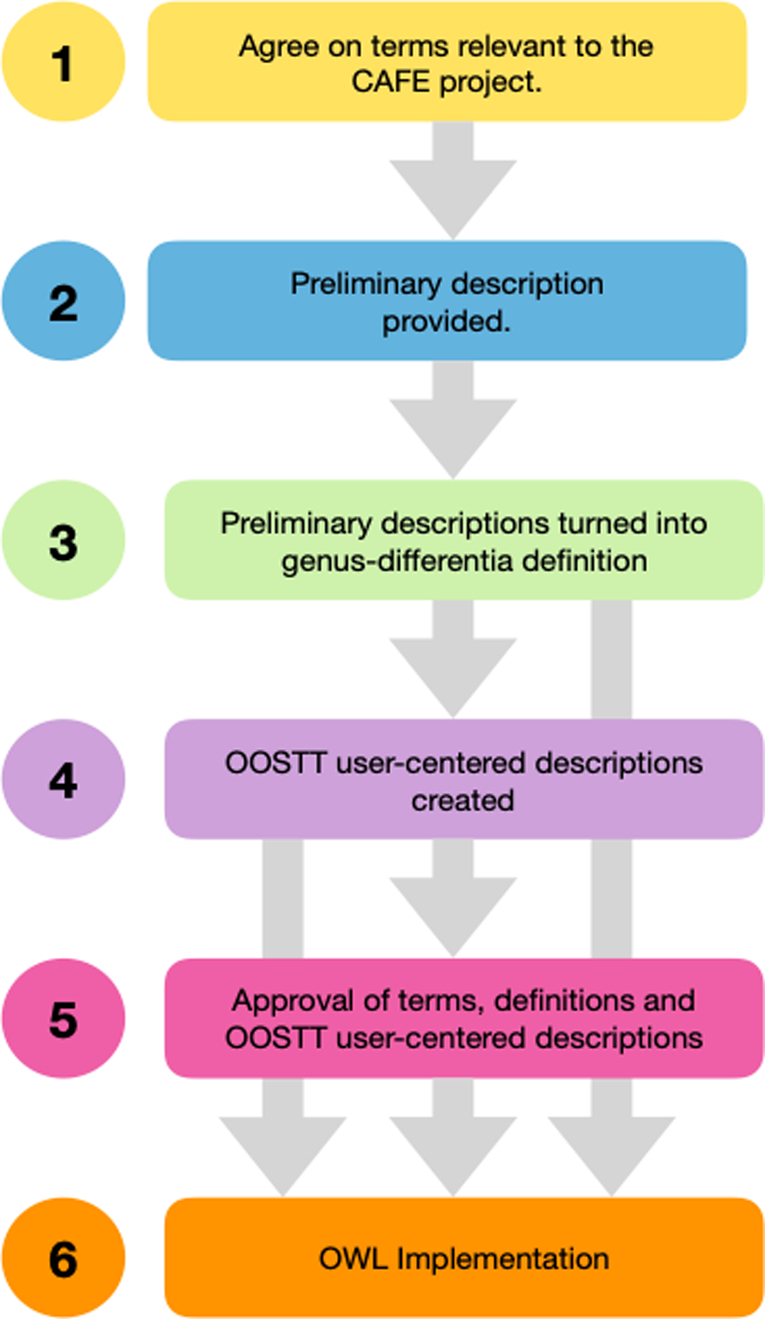
Development workflow of the controlled vocabulary on organizational structures of trauma centers and trauma systems.

**Table 1 –:** Highest rated terms in our controlled vocabulary and their initial definition/OOSTT user-centered description.

Term label	OOSTT user-centered description or definition
trauma program leadership	The individuals who provide oversight, direction, coordination, and management for the facility’s trauma program.
hospital governing body role	A group of individuals appointed to a hospital’s board of directors who provide governance, direction and oversight of the overall operation of a hospital.
trauma program	The organizational unit of a healthcare facility that is designated to provide and coordinate care for injured patients.

**Table 2 –:** Lowest rated terms in our controlled vocabulary and their initial definition/OOSTT user-centered description.

Term label	OOSTT user-centered description or definition
trauma nursing evaluator obligee role	The individual with authority and responsibility for evaluating the nursing care provided to trauma patients.
trauma quality improvement and patient safety program lead role	The role of an authorized group of healthcare providers that ensures trauma quality improvement and patient safety within the trauma program.
emergency medical services provider association	An association of EMS agencies that any agency may join voluntarily to share information or collaborate on issues of state or national importance.

**Table 3 –:** Number of publications retrieved from PubMed using a set of queries related to trauma systems, trauma centers and terminology/vocabulary.

Query	Number of retrieved publications
[“trauma system” terminology]	2
[“trauma center” terminology]	34
[“trauma system” vocabulary]	1
[“trauma center” vocabulary]	1

## Footnotes

1 https://github.com/OOSTT/OOSTT/commit/38c6666824d57da695d38710241495673085b942

2 https://github.com/OOSTT/OOSTT/commit/73f72efc598a813018daaa9397d54657e0ac2107

3 https://github.com/OOSTT/OOSTT/commit/3bcbbfa990e86533068055946e4af21c9833bf71
